# Correlation of Percentage Body Fat, Waist Circumference and Waist-to-Hip Ratio with Abdominal Muscle Strength

**DOI:** 10.3390/healthcare10122467

**Published:** 2022-12-07

**Authors:** Munazza Arif, Davinder K. Gaur, Nishant Gemini, Zaheen A. Iqbal, Ahmad H. Alghadir

**Affiliations:** 1 Banarsidas Chandiwala Institute of Physiotherapy, New Delhi 110019, India; 2 Primus Super Specialty Hospital, New Delhi 110021, India; 3 Department of Rehabilitation Sciences, College of Applied Medical Sciences, King Saud University, Riyadh 11433, Saudi Arabia

**Keywords:** percentage of body fat, waist circumference, waist-to-hip ratio, abdominal muscle strength, bioelectrical impedance, maximum voluntary isometric contraction

## Abstract

Sedentary lifestyle and consumption of high-fat foods have become widespread, especially in the urban population. This leads to a reduction in lean body mass and increased body fat. The correlation between body fat indices and low back pain has been less explored and documented. The aim of this study was to identify the correlation between the percentage of body fat, waist circumference and waist-to-hip ratio and abdominal muscle strength. Percentage of body fat was estimated by using the body composition analyzer method using Tanita BC-545 Innerscan Segmental Body Composition. Waist-to-hip ratio was calculated by dividing the waist circumference by hip circumference. Abdominal muscle (rectus abdominis and external oblique) strength was measured by maximum voluntary isometric contraction as measured by surface electromyography. A positive correlation was observed between waist circumference and the percentage of body fat, while a negative correlation was observed between the average maximum voluntary isometric contraction of rectus abdominis and external oblique muscles and the percentage of body fat. Individuals with a high percentage of body fat tend to have higher fat distribution over the abdominal region and decreased abdominal muscle strength. Therapists should emphasize the use of abdominal muscles in individuals with high body fat in order to reduce the associated risk of the development of poor posture and low back pain.

## 1. Introduction

Fat in the body includes storage and essential fat [1]. Fat accumulation in the adipose tissue is the storage body fat that helps to protect internal organs in the abdomen and chest. Many life and reproductive functions are necessarily maintained by essential body fat. Excess of body fat is referred to as obesity [2]. It is defined by the body mass index (BMI) such that at a given height, higher weight is associated with higher body fat [3]. Since it is not a direct measure of the body’s fat mass and composition, it has often been regarded as an imperfect measure of body fatness in the literature [4,5]. Due to such limitations associated with the BMI, the use of other different measures to assess body fat has been suggested, including the percentage of body fat (PBF), waist–stature ratio (WSR), waist circumference (WC) and waist-to-hip ratio (WHR) [6,7,8,9].

Among women, the percentage of essential body fat is greater as compared to men which has been attributed to the needs of childbearing and other hormonal functions [10]. The PBF is the ratio of total fat mass in a living being to its total body mass. Various methods have been proposed to determine the PBF including measurement with calipers or bioelectrical impedance (BI) analysis. The technique of BI analysis has been reported as a convenient and valid approach for body composition estimation [11,12]. The PBF has been regarded as a measure of fitness level as it is the only measurement that directly calculates relative body composition without regard to weight or height [13]. Similarly, the WHR has been widely used as a person’s health indicator and measure of obesity [14]. It is associated with the risk of developing serious health disorders. A WHR above 0.90 for males and above 0.85 for females has been referred to as abdominal obesity by the World Health Organization [15].

Research shows that body fat distribution is associated with an increased risk of developing diseases, such as coronary artery disease, diabetes, etc., rather than absolute total fat [16,17,18]. Obesity and overweight have been correlated with poor quality of life and musculoskeletal functioning [19]. A major risk factor for developing low back pain (LBP) is being overweight [20]. It has been shown that women with central obesity, as measured by the WC and WHR, have a higher prevalence of LBP further confirming the relationship between body fat distribution and LBP [11,21]. Han et al. also reported that chronic LBP is significantly associated with a high WHR, an indicator of a central obesity pattern, especially in women [21]. Sedentary lifestyle and consumption of high-fat foods have become common, especially in the urban population of the younger age group [22]. This leads to increased body fat and a reduction in lean body mass. This further increase in the PBF has been shown to develop nonspecific chronic LBP and a reduction in aerobic capacity [23]. Patients with nonspecific chronic LBP often report that they have to reduce their level of activity due to pain, creating a vicious cycle. This leads to a long-term reduction in activity levels, developing a ‘deconditioning syndrome’ including weakness of core muscles [24,25,26].

There are fewer studies that study the correlation between LBP and body fat indices [27]. Although an association between abdominal obesity and lower strength of muscles has been shown among older people [28], there are no longitudinal findings that associate body fat or other body composition indices with abdominal muscle strength even in younger age groups or those with normal body weight. Therefore, the objective of this study was to study the correlation between the PBF, WHR and WC and abdominal muscle strength among young people with normal body weight. The outcome of this study shall throw more light on the work of abdominal muscles among individuals with different body fat and help to formulate strategies to decrease the associated risk. In addition, it will further help in understanding the impact of abdominal fat on the reduction in muscle strength.

## 2. Materials and Methods

### 2.1. Study Design

A cross-sectional study design was adopted in this study to identify the correlation between PBF, WC and WHR with abdominal muscles, right rectus abdominis (RA) and right external oblique (EO) strength.

### 2.2. Participants

University students, aged between 18 and 28 years and with a BMI between 18.5 and 24.9 kg/m^2^, were invited to participate in this study through advertisement on the notice board. A total of 50 healthy participants, 36 males and 14 females, were included in this study after careful consideration of the inclusion and exclusion criteria. They were excluded if reported any neurological, musculoskeletal, cardiorespiratory, psychosomatic or hormonal disorders. Subjects who were taking prolonged medications, following a weight training protocol or fasting were not included in the study. This was a one-time study conducted in the university medical center.

### 2.3. Ethical Statement

All the subjects were informed about the procedure, potential risks and benefits of the study. They had to provide written informed consent before data collection. Approval was obtained from the rehabilitation research review board for ethics according to the Declaration of Helsinki guidelines.

### 2.4. Procedure

WC was measured in standing position with the help of a measuring tape at the midway level between the lower rib margin and the iliac crest. It was rounded to the nearest 0.5 cm [29]. The hip circumference (HC) was measured while standing at the widest part of the hip bones of the buttocks region in centimeters to the nearest 0.5 cm [30]. WHR was calculated by ratio of WC and HC [15,31]. Prior to the test the subjects were familiarized with the testing procedure. PBF was estimated by using body composition analyzer method using Tanita BC-545 Innerscan Segmental Body Composition [32,33,34]. The procedure was explained to the participants, and the machine was cleaned using a detergent wipe. In preparation for the analysis, they were asked not to do any exercises or consume alcohol 24 h prior to the test. They were also asked to avoid food or caffeine 4 h before taking the test and to drink 2 to 4 glasses of water 2 h prior to the test. Participants were asked to remove shoes and socks, and their feet were thoroughly wiped using alcohol wipes to clean the area for any cream or moisturizer that could affect the results. They were also asked to remove any outer clothing, empty their pockets and remove any jewelry. The subjects were made to stand barefoot on the footpads on the base of the scale, and a safe weak electric current (50 KHz, 800 uA) was passed through the body via patented pressure contact footpads over a scale platform, and level of resistance was measured in different body segments. They were asked to keep still and look straight ahead while running the test. It was made sure that their arms were extended and any part of body was not under tension. Body composition analyzer has been shown to be a reliable, valid and convenient approach for estimating body composition [11,12,27,35,36,37,38]. The body fat percentage readings using this method are within 5% of DEXA which is the gold standard for body fat measurement.

Abdominal muscle strength was measured by maximum voluntary isometric contraction (MVIC). It is an objective, standardized and sensitive tool for measuring muscle strength [39,40]. For the RA, participants were made to lie in the supine position with hips and knees flexed to 90 degrees and feet supported. They were asked to perform a maximal supine isometric trunk curl-up against manual resistance applied by the therapist at the shoulders in the direction of extension movement to maximally recruit the muscle [40]. Similarly, they were also asked to perform an isometric contraction for EO. Participants had to lie in supine position with hips and knees flexed 90 degrees and feet supported. They were asked to perform isometric contraction against the manual resistance applied by the therapist at the shoulders in the direction of trunk extension and right rotation directions [40]. The muscle bioelectric activity was measured by surface electromyography (EMG) using the 2-channel apparatus Neurotrac^TM^ Myoplus [41,42,43]. Subject skin was prepared by shaving (when necessary) and cleansing it with alcohol before applying electrodes to reduce skin impedance. The EMG signals were recorded in the right RA and EO muscles. Sites for electrode placements for RA were 3 cm lateral to the right of the umbilicus, while for EO, it was approximately 15 cm lateral to the right of the umbilicus [44,45,46]. The bioelectric activity of RA and EO muscles was examined when the subjects performed MVIC for 3 s. All the measurements for all the subjects were conducted by same examiner who was trained for all the procedures before the study was conducted.

### 2.5. Data Analysis

The data were analyzed using SPSS 17 software (SPSS Inc., Chicago, IL, USA). Gender-based differences in PBF, WC, WHR and average MVIC of right RA and EO muscles were analyzed using independent samples t-test. Relationship between PBF, WC, WHR and average MVIC of right RA and EO muscles was determined using Pearson’s correlation coefficients. Correlation was also tested on the basis of gender. The *p*-values less than 0.05 were considered to be significant.

## 3. Results

Demographic characteristics (mean ± SD) and gender-based differences in the PBF, WC, WHR and average MVIC of right RA and EO muscles among the participants are given in Table 1. There were significant differences (*p <* 0.05) in the WHR, PBF and average MVIC of right RA and EO muscles between males and females. Results show a strong positive correlation between WC and the PBF (r = 0.527, *p <* 0.001, 95% CI [0.292, 0.703]) as well as the WHR and WC (r = 0.739, *p <* 0.001, 95% CI [0.580, 0.844]). Among females, there was a strong positive correlation between WC and the PBF (r = 0.757, *p <* 0.001, 95% CI [0.570, 0.869]), WHR and PBF (r = 0.507, *p <* 0.05, 95% CI [0.214, 0.716]) and WHR and WC (r = 0.787, *p <* 0.001, 95% CI [0.619, 0.887]). Among males, there was a strong positive correlation between the WHR and WC (r = 0.614, *p <* 0.05, 95% CI [0.124, 0.864]).

### 3.1. Relation between PBF, WC, WHR and Average MVIC of Right RA

PBF was found to be negatively correlated with the average MVIC of RA (r = −0.329, *p <* 0.05, 95% CI [−0.557, −0.056]). WC (r = 0.043, *p =* 0.76, 95% CI [−0.238, 0.317]) and WHR (r = 0.144, *p =* 0.31, 95% CI [−0.139, 0.406]) were not significantly correlated with the average MVIC of RA. There was no significant correlation (*p >* 0.05) between the PBF, WC, WHR and average MVIC of right RA on the basis of gender.

### 3.2. Relation between PBF, WC, WHR and Average MVIC of Right EO

PBF was found to be negatively correlated with the average MVIC of EO (r = −0.293, *p <* 0.05, 95% CI [−0.529, −0.016]). WC (r = −0.044, *p =* 0.76, 95% CI [−0.319, 0.237]) and WHR (r = 0.106, *p =* 0.46, 95% CI [−0.177, 0.374]) were not significantly correlated with the average MVIC of EO. There was no significant correlation (*p >* 0.05) between the PBF, WC, WHR and average MVIC of right EO on the basis of gender.

## 4. Discussion

This study determined the correlation between the PBF, WC and WHR and abdominal muscle (RA and EO) strength measured by average MVIC. Results show a negative correlation between the PBF and average MVIC of these muscles. This relation was not significant on the basis of gender. Participants with a high PBF exhibited decreased average RA and EO muscle activity.

Overall, there was a positive correlation between the PBF and WC, signifying that participants with a higher PBF had higher fat distribution over the abdominal region. Results further show significant differences in the WHR, PBF and average MVIC of right RA and EO muscles between males and females. Among females, there was a strong positive correlation between the PBF and WC and WHR as well as between the WHR and WC. However, among males, there was a strong positive correlation only between the WHR and WC. As compared to males, various previous studies have reported smaller height, higher weight and differences in the body composition and muscle strength among females [47,48]. The smaller body structure of females acts as a disadvantage during heavy work, lifting weight and when applying body force putting an extra load on their spine [49].

The available literature shows that body fat is more correlated with WC, and the adequacy of the BMI to measure body fat has been doubted [9,37,50,51]. This has led to the development of alternative methods to the BMI when measuring body fat including BI instruments [38,52]. In the BI method, a weak electric current is passed through electrodes placed at different parts of the body, and the level of resistance is measured. The fat-free mass of the body that contains water and electrolytes offers lower resistance, while fat mass provides higher resistance and acts as an isolator [11,12].

The electrical activity of the muscles is recorded by EMG which tests the integrity of the motor system. It shows muscle activation in response to the mobilization of adjoining segments or its participation in a particular movement [53].

Results of previous studies show an inverse relationship between the PBF, physical fitness test scores in tasks such as sit-ups and cardiorespiratory fitness [54,55]. This has been linked to poor abdominal strength and endurance which corroborates with the results of the present study. Fat in the abdominal region affects spinal stability. Studies have linked abdominal obesity with a faster decline in muscle strength among men [28]. Abdominal muscle strength recruitment in individuals with a high PBF may be affected as patients with LBP have been shown to have poor abdominal muscle strength [27,56]. Strong abdominal muscles are required to maintain trunk and spine stability and correct body posture that reduces stress on the lumbar spine [57]. People with an anterior pelvic tilt also often have weak obliques along with other weak muscles including gluteal and tight hip flexors, causing lower back and hip pain [58]. Weak abdominal muscles can cause lower back pain as they encourage a forward-leaning posture and poor stability while doing spinal motions, and focusing rehabilitation only on core muscles is unlikely to fix the problem [59]. In fact, it may further aggravate back pain. As abdominals work in conjunction with back muscles while bending, straightening or lifting, patients are more prone to back pain with a weak core [60,61]. Correct body posture can help in preventing LBP that has been regarded as one of the most common reasons for absence from work [62]. Abdominal muscle strength is also required for good performance in sports activities. Abdominal muscles, including RA and EO, are commonly exercised through concentric muscle contraction causing flexion at the trunk, such as that in a crunch or sit-up exercise, where the resistance is provided only by the moving body mass [63].

Physical activity is necessary for gain in strength, endurance and flexibility [64]. There may be a lack of muscle strength and endurance especially in abdominal muscles among sedentary individuals who have a tendency of obesity [27,65]. This may have a detrimental effect on the lumbar spine, as the abdominal muscles are specifically required to maintain lumbar spine posture [66]. Poor abdominal muscle activation in individuals with a higher PBF, as seen in this study, may lead to the tendency to adopt poor posture. Exercise therapists should emphasize the correct use of abdominal muscles in individuals with high body fat which may in turn reduce the risk of secondary problems associated with sedentary lifestyle.

WC and the WHR are the indirect measures of abdominal fat that have been shown to be highly correlated with the trunk and abdominal visceral fat and regarded as good predictors of abdominal visceral obesity [67]. WHR has been widely used as a person’s health indicator and associated with the risk of developing serious health disorders. These have also been correlated with changes in abdominal visceral fat during weight loss among both men and women [68]. However, there are fewer studies that have studied the relationship between the WHR and muscular strength, and the relationship between abdominal endurance and the WHR has been reported to be not significant [69]. The current study is a correlation study and cannot establish a cause-and-effect relationship between the variables. Future studies should try to establish the relation between the WHR and muscle strength to further explain and classify the MVIC values as poor, normal, moderate or high abdominal strength. Additionally, these studies should also focus on PBF values and how they interpret normal weight, overweight and obesity.

### Limitations

This study consisted of a small number of participants aged 18–28 years and with a BMI of 18.5 to 24.9 kg/m^2^. Surface electromyography includes the limitation of crosstalk. This study should be repeated in different age and ethnic groups to compare abdominal muscle recruitment and anthropometric variables other than the BMI and WHR. We also propose a similar large-scale study in the future to further evaluate such correlation on the basis of gender.

## 5. Conclusions

The results of this study suggest that the PBF has a positive correlation with WC and a negative correlation with RA and EO muscle strength. Participants with a high PBF tend to have higher fat distribution over the abdominal region which may lead to decreased abdominal muscle recruitment. Therapists should emphasize the correct use of abdominal muscles in individuals with high body fat in order to decrease the associated risk of the development of poor posture and LBP.

## Figures and Tables

**Table 1:** Demographic characteristics (mean ± SD) and gender-based differences in PBF, WC, WHR and average MVIC of right RA and EO muscles among the participants (n = 50).

Parameter	Males (n = 14, 28%)	Females (n = 36; 72%)	Total (n = 50)	*p* Value
Age: years	20.28 ± 2.97	21.13 ± 2.20	20.90 ± 2.44	0.272
BMI: kg/m^2^	23.80 ± 1.47	22.46 ± 1.18	23.13 ± 1.32	0.062
Waist circumference (WC): cm	78.35 ± 9.53	75.76 ± 10.57	76.49 ± 10.26	0.428
Hip circumference (HC): cm	89.71 ± 8.71	91.52 ± 8.29	91.02 ± 8.36	0.497
Waist-to-hip ratio (WHR)	0.86 ± 0.04	0.82 ± 0.05	0.83 ± 0.05	**0.015**
PBF	15.34 ± 5.13	25.20 ± 9.33	22.44 ± 9.44	**<0.001**
Average MVIC of right rectus abdominis muscle (mV)	96.79 ± 48.77	33.56 ± 23.31	51.27 ± 42.91	**<0.001**
Average MVIC of right external oblique muscle (mV)	51.09 ± 36.37	21.87 ± 28.20	30.05 ± 33.08	**0.004**

BMI: body mass index, PBF: percentage of body fat, MVIC: maximum voluntary isometric contraction.

## Data Availability

The datasets used in this study are available on reasonable request from the corresponding author.

## References

[1] Kravitz L., Heyward V.H. (1992). Getting a grip on body composition. IDEA Today.

[2] Frankenfield D.C., Rowe W.A., Cooney R.N., Smith J., Becker D. (2001). Limits of body mass index to detect obesity and predict body composition. Nutrition.

[3] Benn R.T. (1971). Some mathematical properties of weight-for-height indices used as measures of adiposity. Br. J. Prev. Soc. Med..

[4] Roche A.F., Sievogel R.M., Chumlea W.C., Webb P. (1981). Grading body fatness from limited anthropometric data. Am. J. Clin. Nutr..

[5] Wellens R.I., Roche A.F., Khamis H.J., Jackson A.S., Pollock M.L., Siervogel R.M. (1996). Relationships between the Body Mass Index and Body Composition. Obes. Res..

[6] Janssen I., Katzmarzyk P., Ross R. (2004). Waist circumference and not body mass index explains obesity-related health risk. Am. J. Clin. Nutr..

[7] Ashwell M., Lejeune S. (1996). Ratio of waist circumference to height may be better indicator of need for weight management. BMJ.

[8] Ashwell M., Hsieh S.D. (2005). Six reasons why the waist-to-height ratio is a rapid and effective global indicator for health risks of obesity and how its use could simplify the international public health message on obesity. Int. J. Food Sci. Nutr..

[9] Flegal K.M., Shepherd J.A., Looker A.C., Graubard B.I., Borrud L.G., Ogden C.L., Harris T.B., Everhart J.E., Schenker N. (2009). Comparisons of percentage body fat, body mass index, waist circumference, and waist-stature ratio in adults. Am. J. Clin. Nutr..

[10] Gallagher D., Heymsfield S.B., Heo M., Jebb S.A., Murgatroyd P.R., Sakamoto Y. (2000). Healthy percentage body fat ranges: An approach for developing guidelines based on body mass index. Am. J. Clin. Nutr..

[11] Toda Y., Segal N., Toda T., Morimoto T., Ogawa R. (2000). Lean body mass and body fat distribution in participants with chronic low back pain. Arch. Intern. Med..

[12] Cha K., Chertow G.M., Gonzalez J., Lazarus J.M., Wilmore D.W. (1995). Multifrequency bioelectrical impedance estimates the distribution of body water. J. Appl. Physiol..

[13] Friedl K., Moore R.J., Martinez-Lopez L.E., Vogel J.A., Askew E.W., Marchitelli L.J., Hoyt R.W., Gordon C.C. (1994). Lower limit of body fat in healthy active men. J. Appl. Physiol..

[14] Elsayed E.F., Tighiouart H., Weiner D.E., Griffith J., Salem D., Levey A.S., Sarnak M.J. (2008). Waist-to-Hip Ratio and Body Mass Index as Risk Factors for Cardiovascular Events in CKD. Am. J. Kidney Dis..

[15] Price G.M., Uauy R., Breeze E., Bulpitt C.J., Fletcher A.E. (2006). Weight, shape, and mortality risk in older persons: Elevated waist-hip ratio, not high body mass index, is associated with a greater risk of death. Am. J. Clin. Nutr..

[16] Perry A.C., Applegate E.B., Allison M.D., Jackson M.L., Miller P.C. (1998). Clinical predictability of the waist-to-hip ratio in assessment of cardiovascular disease risk factors in overweight, premenopausal women. Am. J. Clin. Nutr..

[17] Vismara L., Menegoni F., Zaina F., Galli M., Negrini S., Capodaglio P. (2010). Effect of obesity and low back pain on spinal mobility: A cross sectional study in women. J. Neuroeng. Rehabil..

[18] Deere K.C., Clinch J., Holliday K., McBeth J., Crawley E.M., Sayers A., Palmer S., Doerner R., Clark E.M., Tobias J.H. (2012). Obesity is a risk factor for musculoskeletal pain in adolescents: Findings from a population-based cohort. Pain.

[19] Seidell J., Bakx K.C., Deurenberg P., Burema J., Hautvast J.G., Huygen F.J. (1986). The relation between overweight and subjective health according to age, social class, slimming behavior and smoking habits in Dutch adults. Am. J. Public Health.

[20] Dosta M. (2013). Prevalence of Low Back Pain among the Overweight Person. Bachelor’s Thesis.

[21] Han T., Schouten J.S., Lean M., Seidell J. (1997). The prevalence of low back pain and associations with body fatness, fat distribution and height. Int. J. Obes..

[22] Alghadir A.H., Gabr S.A., Iqbal Z.A. (2016). Television watching, diet and body mass index of school children in Saudi Arabia. Pediatr. Int..

[23] Hodselmans A.P., Dijkstra P.U., Geertzen J.H., van der Schans C.P. (2010). Nonspecific chronic low back pain patients are deconditioned and have an increased body fat percentage. Int. J. Rehabil. Res..

[24] Mayer T.G., Gatchel R.J., Kishino N., Keeley J., Capra P., Mayer H., Barnett J., Mooney V. (1985). Objective Assessment of Spine Function Following Industrial Injury. A prospective study with comparison group and one-year follow-up. Spine.

[25] Kohles S., Barnes D., Gatchel R.J., Mayer T.G. (1990). Improved physical performance outcomes after functional restoration treatment in patients with chronic low-back pain. Early versus recent training results. Spine.

[26] Harriët W., Theresa H.M., Anita W., Andrew S., William R. (2000). Deconditioning in patients with chronic low back pain: Fact or fiction?. Spine.

[27] Nikolov V.T., Petkova M., Kolev N. (2009). Obesity and low back pain in postmenopausal women. J. Biomed. Clin. Res..

[28] de Carvalho D.H.T., Scholes S., Santos J.L.F., de Oliveira C., da Silva Alexandre T. (2019). Does abdominal obesity accelerate muscle strength decline in older adults? Evidence from the English Longitudinal Study of Ageing. J. Gerontol. Ser. A.

[29] Alghadir A.H., Iqbal Z.A., Gabr S.A. (2020). Differences among Saudi and Expatriate Students: Body Composition Indices, Sitting Time Associated with Media Use and Physical Activity Pattern. Int. J. Environ. Res. Public Health.

[30] Alghadir A.H., Gabr S.A., Iqbal Z.A. (2020). Effect of Gender, Physical Activity and Stress-Related Hormones on Adolescent’s Academic Achievements. Int. J. Environ. Res. Public Health.

[31] Alghadir A.H., Gabr S.A., Iqbal Z.A., Al-Eisa E. (2019). Association of physical activity, vitamin E levels, and total antioxidant capacity with academic performance and executive functions of adolescents. BMC Pediatr..

[32] Kolayiş I.E., Arol P. (2020). The effect of Zumba exercises on body composition, dynamic balance and functional fitness parameters in 15-17 years old women with high body mass index. Pedagog. Phys. Cult. Sports.

[33] Ağagündüz D., Gezmen-Karadağ M. (2019). Association of FTO common variant (rs9939609) with body fat in Turkish individuals. Lipids Health Dis..

[34] Ingrová P., Králík M., Bártová V. (2017). Relationships between the hand grip strength and body composition in Czech and Slovak Students. Slov. Antropol..

[35] Raustorp A. (2005). Physical Activity, Body Composition and Physical Self-Esteem among Children and Adolescents.

[36] Gibson A.L., Heyward V.H., Mermier C.M. (2000). Predictive Accuracy of Omron^®^ Body Logic Analyzer in Estimating Relative Body Fat of Adults. Int. J. Sport Nutr. Exerc. Metab..

[37] Deurenberg P., Andreoli A., Borg P., Kukkonen-Harjula K., De Lorenzo A., Van Marken Lichtenbelt W.D., Testolin G., Vigano R., Vollaard N. (2001). The validity of predicted body fat percentage from body mass index and from impedance in samples of five European populations. Eur. J. Clin. Nutr..

[38] Sun S.S., Chumlea W.C., Heymsfield S.B., Lukaski H.C., Schoeller D., Friedl K., Kuczmarski R.J., Flegal K., Johnson C.L., Hubbard V.S. (2003). Development of bioelectrical impedance analysis prediction equations for body composition with the use of a multicomponent model for use in epidemiologic surveys. Am. J. Clin. Nutr..

[39] Meldrum D., Cahalane E., Keogan F., Hardiman O. (2003). Maximum voluntary isometric contraction: Investigation of reliability and learning effect. Amyotroph. Lateral Scler. Other Mot. Neuron Disord..

[40] Lopez-Valenciano A., Biviá-Roig G., Lison-Parraga J., Vera-Garcia F. (2013). Electromyographic study of trunk flexion exercises on inclined board. Rev. Int. Med. Cienc. Act. Fis. Deporte.

[41] van Vuuren E.C.J., Brandt C. (2019). An International Classification of Function, Disability and Health (ICF)-based investigation of movement impairment in women with pelvic organ prolapse. S. Afr. J. Physiother..

[42] Boguszewski D., Oko B., Adamczyk J.G., Bialoszewski D. (2016). Evaluation of the effectiveness of kinesiotaping in reducing delayed onset muscle soreness of the biceps brachii. Biomed. Hum. Kinet..

[43] Ojukwu C.P., Ikele C.N., Nwobodo O.D., Okemuo A.J., Ikele I.T., Uchenwoke C.I., Ezeugwu U.A. (2020). Electromyographic activity of the neck muscles: Effects of varying standing height-derived teaching board heights. J. Back Musculoskelet. Rehabil..

[44] Kesavachandran C., Bihari V., Mathur N. (2009). Can physical activity maintain normal grades of body mass index and body fat percentage?. Int. J. Yoga.

[45] García-Vaquero M.P., Moreside J.M., Brontons-Gil E., Peco-González N., Vera-Garcia F.J. (2012). Trunk muscle activation during stabilization exercises with single and double leg support. J. Electromyogr. Kinesiol..

[46] Bouillon L.E., Wilhelm J., Eisel P., Wiesner J., Rachow M., Hatteberg L. (2012). Electromyographic assessment of muscle activity between genders during unilateral weight-bearing tasks using adjusted distances. Int. J. Sports Phys. Ther..

[47] Shehab D., Al-Jarallah K., Moussa M.A., Adham N. (2003). Prevalence of Low Back Pain among Physical Therapists in Kuwait. Med. Princ. Pract..

[48] Miller A.E.J., MacDougall J., Tarnopolsky M., Sale D. (1993). Gender differences in strength and muscle fiber characteristics. Eur. J. Appl. Physiol. Occup. Physiol..

[49] Alghadir A., Zafar H., Iqbal Z.A. (2015). Work-related musculoskeletal disorders among dental professionals in Saudi Arabia. J. Phys. Ther. Sci..

[50] Lean M.E., Han T., Deurenberg P. (1996). Predicting body composition by densitometry from simple anthropometric measurements. Am. J. Clin. Nutr..

[51] Bosy-Westphal A., Geisler C., Onur S., Korth O., Selberg O., Schrezenmeir J., Müller M.J. (2006). Value of body fat mass vs anthropometric obesity indices in the assessment of metabolic risk factors. Int. J. Obes..

[52] Lintsi M., Kaarma H., Kull I. (2004). Comparison of hand-to-hand bioimpedance and anthropometry equations versus dual-energy X-ray absorptiometry for the assessment of body fat percentage in 17-18-year-old conscripts. Clin. Physiol. Funct. Imaging.

[53] World Health Organization (2011). Waist Circumference and Waist-Hip Ratio: Report of a Who Expert Consultation, Geneva, 8–11 December 2008.

[54] Escamilla R.F., Lewis C., Bell D., Bramblet G., Daffron J., Lambert S., Pecson A., Imamura R., Paulos L., Andrews J.R. (2010). Core Muscle Activation during Swiss Ball and Traditional Abdominal Exercises. J. Orthop. Sports Phys. Ther..

[55] Kiflu A., Reddy R., Babu M.S. (2012). Relationship of body fat percentage and selected physical fitness performances between overweight and normal weight sedentary young male adults. Res. J. Recent Sci..

[56] Massó N., Rey F., Romero D., Gual G., Costa L., Germán A. (2010). Surface electromyography applications in the sport. Apunt. Med. Esport.

[57] Lee K. (2021). The Relationship of Trunk Muscle Activation and Core Stability: A Biomechanical Analysis of Pilates-Based Stabilization Exercise. Int. J. Environ. Res. Public Health.

[58] SF Mace; SF Kettlebell; SF Band. What Is Anterior Pelvic Tilt & How Do You Fix It?. https://www.setforset.com/blogs/news/anterior-pelvic-tilt.

[59] Gluppe S., Engh M.E., Kari B. (2021). Women with diastasis recti abdominis might have weaker abdominal muscles and more abdominal pain, but no higher prevalence of pelvic floor disorders, low back and pelvic girdle pain than women without diastasis recti abdominis. Physiotherapy.

[60] Ershad N., Kahrizi S., Abadi M.F., Zadeh S.F. (2009). Evaluation of trunk muscle activity in chronic low back pain patients and healthy individuals during holding loads. J. Back Musculoskelet. Rehabil..

[61] Kato K., Otoshi K.-I., Yabuki S., Otani K., Nikaido T., Watanabe K., Kobayashi H., Handa J.-I., Konno S.-I. (2020). Abdominal oblique muscle injury at its junction with the thoracolumbar fascia in a high school baseball player presenting with unilateral low back pain. Fukushima J. Med. Sci..

[62] Alghadir A., Zafar H., Iqbal Z.A., Al-Eisa E. (2017). Work-Related Low Back Pain Among Physical Therapists in Riyadh, Saudi Arabia. Work. Health Saf..

[63] Brown G.A., Heiserman K., Shaw B.S., Shaw I. (2015). Rectus abdominis and rectus femoris muscle activity while performing conventional unweighted and weighted seated abdominal trunk curls. Med. Sport.

[64] Ferreira M.L., Sherrington C., Smith K., Carswell P., Bell R., Bell M., Nascimento D.P., Pereira L.S.M., Vardon P. (2012). Physical activity improves strength, balance and endurance in adults aged 40–65 years: A systematic review. J. Physiother..

[65] Skrypnik D., Bogdański P., Mądry E., Karolkiewicz J., Ratajczak M., Kryściak J., Pupek-Musialik D., Walkowiak J. (2015). Effects of Endurance and Endurance Strength Training on Body Composition and Physical Capacity in Women with Abdominal Obesity. Obes. Facts.

[66] McGill S.M., Grenier S., Kavcic N., Cholewicki J. (2003). Coordination of muscle activity to assure stability of the lumbar spine. J. Electromyogr. Kinesiol..

[67] Nunes P.R.P., Barcelos L.C., Oliveira A.A., Júnior R.F., Martins F.M., Orsatti C.L., Resende E.A.M.R., Orsatti F.L. (2016). Effect of resistance training on muscular strength and indicators of abdominal adiposity, metabolic risk, and inflammation in postmenopausal women: Controlled and randomized clinical trial of efficacy of training volume. AGE.

[68] Paré A., Dumont M., Lemieux I., Brochu M., Alméras N., Lemieux S., Prud’homme D., Després J.-P. (2012). Is the relationship between adipose tissue and waist girth altered by weight loss in obese men?. Obes. Res..

[69] Islam M.S. (2018). Relationship of abdominal muscle endurance with selected anthropometric measurements in soccer players. Int. J. Physiol. Nutr. Phys. Educ..

